# Incentivising participation in mental health app research: lessons learned from a mixed methods randomised controlled trial

**DOI:** 10.1192/bjo.2025.48

**Published:** 2025-05-19

**Authors:** Holly Alice Bear, Catherine Money, Edward Watkins, Mina Fazel

**Affiliations:** Department of Psychiatry, University of Oxford, Oxford, UK; School of Psychology, University of Exeter, Exeter, UK

**Keywords:** Mental health apps, randomised controlled trial, engagement, young people, implementation

## Abstract

**Background:**

User engagement is recognised as a critical and pervasive challenge that has limited the potential evidence base being developed for mental health apps.

**Aim:**

To understand young people’s motivations for participating in a randomised controlled trial for a mental health app and the role of intrinsic (e.g. improving well-being) and extrinsic (e.g. financial incentives) drivers in engagement.

**Method:**

Emotional Competence for Well-Being (ECoWeB) was a superiority parallel three-arm randomised cohort trial recruiting a cohort of 16–22 year-olds across the UK, Germany, Spain and Belgium, who, depending on risk, were allocated respectively to the PREVENT (*n* = 1262) versus PROMOTE (*n* = 2532) trials. We conducted in-depth semi-structured interviews in the UK (*n* = 18, mean age = 17.7, s.d. = 1.5) and Spain (*n* = 11, mean age 20.6, s.d. = 1.7) to explore participants’ self-reported motivations and engagement. The trial was registered at ClinicalTrials.gov: NCT04148508.

**Results:**

Across arms, 21% of participants never set up an account to access the app and approximately 50% did not complete the 3-month follow-up assessment. Engagement was not significantly higher in the intervention arm compared to the control arms across metrics. Qualitative findings demonstrated that although extrinsic factors alone may be enough to prompt someone to sign up to research, intrinsic drivers (e.g. finding the app useful) are needed to ensure longer-term engagement.

**Conclusions:**

Incentivising participation in clinical trials needs to be consistent with incentives that might be utilised at the point of dissemination and implementation to ensure that findings are replicated if that intervention is adopted at scale.

Mental health applications (‘apps’) have been suggested as a possible solution to some of the complex healthcare barriers affecting populations not accessing services in the expected numbers – especially younger populations. Given that smartphone ownership is nearly ubiquitous among young people in high-income nations and increasingly across lower-resource settings, apps have the potential to address some of the accessibility and scalability issues in service provision for young people’s mental health.^1^ In response, there has been a rapid expansion in the number of apps being developed, both commercially and in academic research programmes, which aim to improve mental health and well-being. Recent estimates suggest that anywhere from 10 000 to 22 750 mental health apps exist.^2,3^ Some apps are integrated into clinical services, supplementing traditional interventions, and emerging evidence from trials indicates that certain apps can lead to significant symptom improvement compared to control conditions.^4–7^

Despite the rapid growth in mental health app development, few have openly published real-world data to support their effectiveness, and even fewer have been successfully implemented as sustainable interventions in practice.^8–12^ Many apps can be accessed directly by young people in the commercial marketplace as self-care tools, but most lack sufficient empirical evidence to justify their use.^13,14^ This gap between app availability and evidence-based implementation underscores a critical challenge: achieving and sustaining user engagement, both within trials and in real-world settings. Poor engagement significantly limits the ability to assess the effectiveness of these interventions, with real-world app retention rates often dropping below 5% after just 30 days.^15^ Understanding the factors that drive young people’s participation in trials and their engagement with mental health apps is therefore critical for optimising trial designs and improving scalability in real-world contexts.

One of the most pressing challenges facing the study and successful implementation of mental health apps, especially when provided as an unguided, standalone intervention, is that recruitment is often financially incentivised, highly prescriptive and unrepresentative in trials, thus reducing the external validity of findings and hampering future scalability efforts. Even with financial incentives, a key challenge is establishing and maintaining user engagement with the intervention.^8,10,13,16,17^ Research suggests that the majority of those offered these app-based interventions do not engage at the recommended frequency or complete the full course of treatment.^9^ Real-world objective data on user engagement with popular mental health apps show general user retention is poor, with a median 15-day retention of 3.9% and 30-day retention of 3.3%.^15^ App-based intervention studies face similar barriers to engagement. In a recent review of 100 000 users’ engagement trajectories across different remote digital health studies, including for depression, median app engagement was only on 5.5 days in the first 12 weeks.^18^ The reporting of intervention engagement in trials is highly variable and a number of basic metrics of intervention engagement, such as rate of intervention uptake, intervention adherence, weekly use patterns and number of intervention completers, are available yet often not routinely reported.^9^ As a result, few apps prove successful in the crowded and unregulated health app market, with only a small number building a regular userbase.^14^ As a whole, there is little evidence that mental health apps can effectively engage young people over a sustained period of time.^19^ Understanding engagement drivers may inform efforts to develop more effective and appealing mental health apps and ensure that they reach wider audiences.

Poor engagement is likely driven by several factors that include, but are not limited to, interventions not being user-friendly, not being designed in a user-centric manner, low intrinsic motivation to participate, poor intervention acceptability and feasibility, technical difficulties, lack of intervention credibility, concerns about privacy and being perceived as an untrustworthy source of mental health information.^10,15,20,21^ Even with the use of recruitment advertisements (e.g. posters and flyers), non-financial behavioural incentives (e.g. motivational messaging and gamification) and financial rewards (e.g. shopping vouchers and prize draws), incentives have often been insufficient to generate and retain sufficient participation and retention in app-based intervention research.^8^ Although the use of financial incentives can improve rates of retention and recruitment incentivising participation,^22,23^ the validity of findings on intervention engagement and the role of internal motivators can be compromised.^24,25^

While financial incentives are commonly employed in research to improve recruitment and retention, their role in fostering sustained engagement with mental health apps remains underexplored.^26^ Specifically, there is limited understanding of how these extrinsic motivators, such as shopping vouchers provided as reimbursement for time spent completing assessments, compare to intrinsic factors, such as the app’s perceived usefulness and relevance to the user’s personal goals. These financial incentives also differ from gamification rewards embedded within the app, which are designed to enhance engagement by leveraging psychological mechanisms, such as reward systems potentially involving dopamine pathways associated with motivation and reinforcement.^27^ In addition, little evidence exists on whether the incentives that are effective during research trials can translate seamlessly to broader implementation at scale. These gaps underscore the need for mixed-methods research to investigate the dynamic interplay between intrinsic and extrinsic motivators.

Such insights are crucial for optimising trial designs to align more closely with real-world adoption scenarios, ensuring that findings are both replicable and impactful. Furthermore, there is a notable lack of research on the specific challenges and motivators associated with engaging young people in mental health app trials. Addressing these issues is essential for improving both recruitment and engagement, thereby enhancing the validity and applicability of trial outcomes. While trial participation and real-world engagement are distinct, exploring these dynamics in a controlled trial setting provides valuable insights into factors that may enhance engagement with mental health apps at scale. Addressing these issues is essential for optimising trial designs and ensuring that findings translate effectively to real-world contexts.

## Research questions

Using mixed methods, the primary aim of this research was to understand young people’s motivations for enrolling and participating in a mental health app trial, particularly the role of intrinsic (e.g. improving well-being) and extrinsic (e.g. financial incentives) drivers in motivating their effective intervention engagement.

- What are the rates of effective engagement in the trial, including study recruitment, retention, attrition and app use? Do these differ between intervention groups?
- What are young people’s motivations for enrolling and participating in the trial and their self-reported engagement?

## Method

### Study design

The Emotional Competence for Well-Being (ECoWeB) trial was a cohort multiple randomised controlled trial (cmRCT) recruiting a cohort of young people (aged 16–22) across the UK, Germany, Spain and Belgium. Participants were allocated into one of two superiority parallel three-arm randomised multicentre, multinational trials based on their assessed risk for future poor mental health: the PREVENT trial for those at high risk and the PROMOTE trial for those at low risk.^28–30^ The trial was conducted between October 2020 and August 2022.

We also conducted in-depth semi-structured interviews in the UK and Spain to explore trial participants’ motivations for participating in the trial, self-reported engagement and views about the intervention. Interviews were conducted between July and December 2021. Ethical approval was provided by each site’s respective institutional research ethics boards. Additional ethical approvals for the qualitative interviews were granted, in the UK, by the University of Exeter Research Ethics Committee (eCLESPsy000048 v10.0), and in Spain, by the Jaime I University Research Ethics Committee (CD/93/2021). The authors assert that all procedures contributing to this work comply with the ethical standards of the relevant national and institutional committees on human experimentation and with the Helsinki Declaration of 1975, as revised in 2013. Full details of the methods and results of the trial, including the CONSORT checklist and outcomes, are presented in full in Watkins et al.^29,30^

### Intervention

The PREVENT trial compared the efficacy of the emotional competence intervention (emotional competence app + usual care) to a generic cognitive–behavioural therapy (CBT) intervention (CBT app + usual care) and a self-monitoring control arm (self-monitoring app + usual care) for participants at high risk of poor mental health. The PROMOTE trial similarly compared these three intervention arms – emotional competence app, CBT app and self-monitoring app – in participants at low risk of poor mental health. Each intervention arm offered specific features through the app, MyMoodCoach.

- Control arm (self-monitoring-app + usual care): access to self-monitoring features (daily mood ratings, diary options, progress dashboards), plus any additional usual care a participant may receive external to the trial.
- Active control arm (CBT app + usual care): access to self-monitoring features, usual care and generic CBT self-help strategies.
- Active experimental arm (emotional competence app + usual care): access to self-monitoring features, usual care and personalised emotional competence strategies targeting emotion regulation and emotional knowledge.

The emotional competence intervention was designed to target certain processes potentially affecting mental well-being, including, but not limited to, improving emotion regulation by reducing maladaptive strategies such as worry and rumination and replacing them with constructive alternatives and problem-solving. It also tried to enhance emotional knowledge and perception through psychoeducation and app-based learning tasks. All versions included self-monitoring (regular daily mood rating, diary option); ecological momentary assessments for more detailed analysis of mood, activity and situational context; a menu structure including a dashboard to monitor progress; and an explore function to graph the self-monitoring responses made by the participant.

The two active intervention arms (emotional competence app, CBT app) also included ‘Challenges’ that provide psychoeducation and learning exercises and ‘Tools’ that are brief strategies that young people can use in the moment when they need them. Challenges and Tools included text, pictures, animated videos, audio-exercises to practise, questionnaires with tailored feedback and quizzes. To try and increase adherence to the app, completion of self-monitoring, ‘Challenges’ and ‘Tools’ were each gamified, with badges earned for adherence and progress.

### Incentives

#### Intrinsic

Participants were given access to a free app containing a variety of psychoeducational tools and self-help strategies that may help improve their mental well-being.

#### Extrinsic

Participants were paid in electronic shopping vouchers for taking part in the three follow-up assessments (£10 for completing each of the three follow-ups). Participants were also able to gain badges that they could exchange for rewards through the gamification system of the app. The gamification system on the app rewarded the earning of badges at different levels (complete all available badges at bronze earn £10; complete all at silver earn £10; complete all at gold earn £10). Participants could therefore earn up to a maximum of £60 for taking part in the trial. European participants were paid the equivalent in Euros.

### Trial participant recruitment

Eligibility inclusion criteria were (a) aged 16–22 years old, (b) living in the UK, Germany, Spain or Belgium, (c) having basic literacy in at least one study language, (d) able to provide informed consent and obtain parental consent for those aged under 18 years old in Germany and Belgium, (e) having regular access to a smartphone (Android or iOS) and (f) elevated hypothesised vulnerability on emotional competence profile based on baseline assessment of emotional competence skills (PREVENT). Participants were recruited across the UK, Germany, Spain and Belgium via online and website advertising; a social media and press campaign; newsletters and other circulars; and notice boards within willing schools, colleges and universities. Following pre-screening there was a date of birth check to ensure the participant was eligible for the trial and if they need parental permission to consent to take part in the trial (Germany or Belgium). Those needing parental permission were asked to complete their parents’ contact details and an email was automatically sent to their parents with information about the trial and a unique link for them to give parental permission for their child. After parental consent was given, the participant was automatically sent an email link to return to the consent page. Participants provided written electronic informed consent. Gender data was collected via self-report, with participants asked to identify their gender. For full details on participants’ mental health profiles and associated baseline measures, please refer to Watkins et al.^29,30^

### Statistical analysis

All participants were followed up at 1, 3 and 12 months post-randomisation. Adherence was defined *a priori* from the logic model for the therapy (see Supplementary Fig. 1 in Watkins et al^29^) and the associated gamification for the app. Retention rates were calculated based on the number of participants completing follow-up assessments at 1, 3 and 12 months. Primary analyses compared the three treatment groups (self-monitoring, CBT, emotional competence) for both trials (PREVENT and PROMOTE) and used collected app engagement data (e.g. the number of days that the app and self-monitoring were used) at the 3-month follow-up, using hierarchical linear regression models with adjustment for, age, gender and country. Three regressions were performed for total app use, overall number of days self-monitoring used and overall number of days app used. Statistical significance was set at *p* < 0.05, and results were interpreted alongside effect sizes to provide a comprehensive understanding of the findings.

### Qualitative sub-study

#### Participant recruitment

Participants in the UK and Spain were emailed a letter of invitation to participate in a follow-up interview. Those invited to participate had to meet the following criteria: (a) had previously agreed to be contacted during the informed consent process of the trial, (b) were randomised to be in the emotional competence app condition, (c) had downloaded the app and (d) had been enrolled in the trial for 3 months. The email contained a hyperlink for those who wished to take part, which led to a Qualtrics page containing the information sheet and consent form, privacy notice, additional embedded signposting and safeguarding materials and a set of short questionnaires from which to gather data on baseline demographics and mood, for example, age, gender, ethnicity, educational attainment and nationality, as well as questions on their mood. Consenting participants were then asked to provide contact details for a researcher to contact them to arrange the interview.

#### Participants

**Fig. 1:**
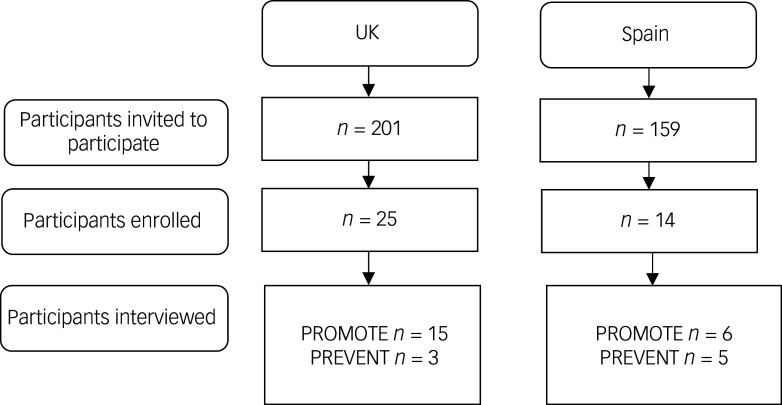
Participant recruitment for the qualitative sub-study.

We interviewed 29 trial participants in total, including 18 in the UK (mean age = 17.7, s.d. = 1.5) and 11 in Spain (mean age 20.6, s.d. = 1.7) (Fig. 1). In the UK, 89% of those interviewed were female compared to 73% in Spain (Table 1). Some 21/29 participants were in the PROMOTE trial in the UK (*n* = 15) and in Spain (*n* = 6) (18/21 female, mean age = 18.6 years). Some 8/29 participants were in the PREVENT trial in the UK (*n* = 3) and in Spain (*n* = 5) (6/8 female, mean age = 19.4 years).

**Table 1:** Participant characteristics

Charactertistics	UK (*n* = 18)	Spain (*n* = 11)	PREVENT (*n* = 8)	PROMOTE (*n* = 21)
Gender				
Female	16 (89)	8 (73)	6 (75)	18 (86)
Male	2 (11)	3 (27)	2 (25)	3 (14)
Age	17.7 ± 1.5	20.6 ± 1.7	19.4 ± 2.6	18.6 ± 2.0
Ethnicity				
White	13 (72)	9 (82)	7 (88)	15 (71)
Black	2 (11)	0 (0)	0 (0)	2 (10)
Mixed/multiple ethnicities	2 (11)	2 (18)	1 (12)	3 (14)
Prefer not to answer	1 (6)	0 (0)	0 (0)	1 (5)
Refugee or an asylum seeker	0 (0)	0 (0)	0 (0)	0 (0)
Chronic medical condition	0 (0)	0 (0)	0 (0)	0 (0)
Disability	3 (17)	0 (0)	3 (38)	0 (0)
Educational attainment				
Lower secondary school	11 (61)	1 (9)	4 (50)	7 (33)
Upper secondary school	7 (39)	5 (46)	2 (25)	11 (52)
Other higher education	0 (0)	2 (18)	1 (12)	1 (5)
Undergraduate degree	0 (0)	3 (27)	1 (12)	2 (10

#### Procedure

Participants provided written informed consent, followed by demographic information, and answered structured questions about their use of mental health services and comorbid mental and physical health conditions online. Interviews were conducted by postdoctoral researchers, author H.A.B. (UK) and independant researcher L.A.N. (Spain), on Microsoft Teams (version 25044.2208.3471.2155) and lasted approximately 45 min. Interviews followed a semi-structured schedule based on a taxonomy of implementation outcomes.^31^ Topics included self-reported app usage, motivations for participating in the study, perceived positive and negative impact of using the app and feedback about the app. With prior consent, all interviews were audio-recorded. All participants were incentivised to participate with a £/€20 shopping voucher.

#### Analytic strategy

Interviews were transcribed verbatim, and the transcripts were assigned a unique pseudonym to anonymise participants. Transcripts from interviews conducted in Spain were translated from Spanish to English by researcher L.A.N. The interviews were analysed using a combination of theory- and data-driven analysis techniques, consisting primarily of deductive, theory-driven thematic analysis.^32^ Analysis of the transcripts in the UK and Spain was conducted by H.A.B., using NVivo11 for Windows (QSR International Pty Ltd, Melbourne, Australia; see https://www.qsrinternational.com). Initial familiarisation with the data was achieved through the transcription process and iterative re-reading of the interviews. Analysis was carried out through a recursive process of open coding, when concepts were named and their properties and dimensions identified, followed by axial coding, when links and associations were drawn between codes. Codes were based on language used by the young people and were applied to each new unit of meaning. Data extracts were multiple coded when appropriate, as were contradictory and minority features of the data. The data-set was iteratively reviewed, and codes were systematically applied to the whole data-set until a finalised coding manual was established. Codes were organised into potential themes using thematic maps and tables. We then compared the content of themes between the PROMOTE and the PREVENT trial participants. The development of the coding manual was iteratively reviewed and refined through discussion with researcher L.A.N. and author M.F. throughout the analysis process to ensure the reliability and rigour of the process and results.

## Results

### Quantitative findings

#### Recruitment and retention

Between 15 October 2020 and 3 August 2021, 21 277 individuals visited the online screener for the ECoWeB cohort, 10 030 accessed the baseline assessment and 3794 were eligible for inclusion in the ECoWeB cohort, of which 1262 were consented for the ECoWeB-PREVENT trial and were randomly allocated (emotional competence app *n* = 417; CBT app *n* = 423; self-monitoring app *n* = 422). The number of participants not completing follow-up assessments at 1, 3 and 12 months was 633 (50%), 699 (55%) and 726 (57.5%), respectively. A further 2532 were consented for the ECoWeB-PROMOTE trial (those with lower vulnerability on the emotional competence measures taken at baseline). Eligible participants were randomly allocated (emotional competence app *n* = 847; CBT app *n* = 841; self-monitoring app *n* = 844). The number of participants not completing follow-up assessments at 1, 3 and 12 months was 1151 (46.5%), 1217 (48.1%) and 1272 (50.2%), respectively. Across all three arms, 21% of participants never set up an account to access the app. Participant demographic data are presented in Table 2. For a detailed flowchart illustrating participant flow from screening to follow-up, please refer to Watkins et al.^29,30^

**Table 2:** Participant demographic data

Demographics	PREVENT	PROMOTE
Age, mean (s.d.)	18.82 (1.99)	19.03 (1.90)
Country		
England	294	559
Spain	305	312
Germany	168	716
Belgium	59	155
Gender		
Male	140	336
Female	667	1392
Neither	12	9
Both	7	5
Trial arm		
Self-monitoring app	285	577
CBT app	273	579
Emotional competence app	268	586

CBT, cognitive–behavioural therapy

#### Intervention adherence

Participant engagement data are presented in Table 3. At the 3-month follow-up, after controlling for age, gender and country, trial condition explained a small but significant proportion of variance in the overall number of days the app was used in both the PREVENT, *R* ^2^ = 0.21, *F*(1, 800) = 216.3, *p* < 0.001, and PROMOTE trials, *R* ^2^ = 0.16, *F*(1, 1709) = 315.6, *p* < 0.001. Trial condition did not explain a significant proportion of variance in total app use in the PREVENT trial, *R* ^2^ = 0.0, *F*(1, 818) = 0.0, *p* = 0.99, or PROMOTE trial, but country did explain a very small but significant proportion of variance in the PROMOTE trial, *R* ^2^ = 0.01, *F*(1, 1735) = 11.59, *p* < 0.001. Finally, age explained a very small but significant proportion of variance in the number of days self-monitoring was used in the PREVENT trial, *R* ^2^ = 0.008, *F*(1, 818) = 6.76, *p* < 0.01, and country explained a small but significant proportion of variance in the PROMOTE trial, *R* ^2^ = 0.03, *F*(1, 1735) = 58.3, *p* < 0.001.

**Table 3:** Participant engagement in each arm (3-month follow-up)

Trial arms	Engagement outcomes					
	Number of days self-monitoring used	Overall number of days app used	Emotion wheel from the emotion monitor	Total app use	Total Tool use	Total Challenge use
Self-monitoring app	13.15 (19.42)	31.89 (14.18)	13.15 (19.43)	129.64 (52.52)	N/A	N/A
CBT app	13.30 (21.73)	51.52 (18.4)	13.30 (21.73)	134.10 (60.89)	2.55 (4.73)	2.42 (2.61)
Emotional competence app	12.41 (19.76)	51.23 (17.1)	12.41 (19.76)	130.53 (57.18)	2 (4.28)	1.59 (1.84)
Total	12.95 (20.32)	44.81 (19.1)	12.95 (20.32)	131.42 (56.96)	1.51 (3.84)	1.33 (2.1)

CBT, cognitive–behavioural therapy

Estimated marginal means (EMMs) were calculated to compare overall number of days of app use across the three study groups (CBT, emotional competence and self-monitoring), adjusting for age, gender and country. In the PREVENT trial, pairwise comparisons revealed that participants in the CBT group (adjusted mean = 53.8, s.e. = 0.97) had significantly higher engagement levels than those in the self-monitoring group (adjusted mean = 31.4, s.e. = 0.97; *p* < 0.001). Similarly, the emotional competence app group (adjusted mean = 52.48, s.e. = 0.98) demonstrated significantly higher engagement compared to the self-monitoring group (*p* < 0.001), but no significant difference was observed between the CBT app and emotional competence app groups (*p* = 1.0).

Similarly, in the PROMOTE trial, pairwise comparisons revealed that participants in the CBT app group (adjusted mean = 50.57, s.e. = 0.71) had significantly higher engagement levels than those in the self-monitoring group (adjusted mean = 32.1, s.e. = 0.71). Similarly, the emotional competence app group (adjusted mean = 50.7, s.e. = 0.71) demonstrated significantly higher engagement compared to the self-monitoring group (*p* < 0.001), but no significant difference was observed between the CBT app and emotional competence app groups (*p* = 1.0).

### Qualitative findings

#### Self-reported motivation

In term of participants’ motivations for taking part in the research and motivation for engagement, participants cited a variety of intrinsic (i.e. internal factors such as personal satisfaction and enjoyment without gaining external rewards) and extrinsic (i.e. external factors such rewards or other incentives – such as praise, course credits or money) motivating factors that promoted them to sign up for the study. Often it was a combination of factors that motivated people to participate, spanning both extrinsic and intrinsic drivers. Although extrinsic factors alone, for example, financial incentives, may be enough to prompt someone to sign up to and engage in research, intrinsic drivers are needed to ensure longer term engagement and participation. The most important extrinsic driver was the financial incentive, which was seen as relatively accessible and easy to earn. Some young people participated because they were studying psychology and seemed motivated to gain research experience (intrinsic) or needed course credits from completing research studies (extrinsic). Finally, some people were driven by the badges and the gamification of the app:

*‘The vouchers. Yeah. I’ve done so many of those. Yeah, it’s the incentive. I’ve done so many. Uh, yeah it was just the badges to be honest. The badges and obviously these like equate to the vouchers. As I said before, yeah.’* UK

*‘Well, I’m a psychology student at X University so they sent out an email about it. I’m trying to take part in as many studies as I can. Yeah, mainly on to gain experience and how it all works.’* UK

*‘(…) one of the things that led me to use the app were also the payments, that they are there and for doing easy things I thought it was nice.’* Spain

The financial incentive was a prominent extrinsic driver, but some people were intrinsically motivated too. The most commonly mentioned intrinsic motivating factors included the desire to help others, curiosity, general interest in the research topic and the desire to improve one’s own mental health. The within-app notifications served as a useful prompt to remind people that the app was on their phone:

*‘And I just thought it sounded like a really interesting study, and it was like targeting my age group. So, I thought it would be nice to be like involved and see how it could help me and like how it could help other young people. And I quite liked the idea of the emotion monitor, and also, I quite enjoyed looking at the different tools. And just like the functions which are available on the app.’* UK

#### Self-reported engagement

A subset of young people were very committed to using the app regularly and appeared highly motived and engaged throughout. However, a consistent finding was that most young people tended to use the app most when they first downloaded it, with a gradual reduction in usage over time:

*‘At the beginning, when I downloaded it I did use it almost every day, also because I had the notification on and therefore I could analyse a bit how I had felt throughout the day (…) But I changed phones and I did not set up the alarm on this mobile phone (…) Perhaps I would enter the app once a week, then less and now I hadn’t use it for maybe a couple of weeks.’* Spain

*‘I used it more at the beginning than I do now, but yes, I have been using it these months (…) at the beginning I used it practically every day. I think I spent a month, a little over a month, using it almost daily and then it was twice a week. And I am still using it like that.’* Spain

*‘So, at the beginning I was using it all the time every day, like for the first few weeks…. I think because it was quite time-consuming eventually it started like I started using it less and less. Now, to be honest, I barely use it because this demotivated me, I guess.’* UK

As part of the interview, we asked participants if they would have used the app less if they were not being paid to use it. Responses to this question varied, with some young people saying that the payment was not important to them and that they would have used it the same amount and others reporting that they would likely have used it less frequently. This finding highlights the importance of individual intrinsic motivation in app usage, for example, different drivers are important to different people:

*‘Maybe a little bit, yeah. But I think it still would have been helpful even if there wasn’t the monetary reward.’* UK

*‘Yeah, I don’t think I would have used it. I probably would have used it maybe a little bit, but yeah, if the badges weren’t there, probably not nearly as much, as I have used it already.’* UK

*‘I think in its current state I probably would have used it less because I don’t really like the overall interface of that app, but if it was improved, I think I would have used it without the incentive of vouchers.’* UK

#### Payment model preferences

Most participants reported that they would not pay for the app in its current form. One of the reasons for this was that they did not have the money to spare, whilst others would be more likely to pay if it could offer interactions with professionals. Participants widely understood the need for an app to be financed, and therefore on the whole said they would tolerate non-invasive adverts with the ability to purchase a premium ad-free version. Some participants were particularly opposed to the suggestion of disruptive, full-page video adverts, citing that these unpredictable forms of advertising can cause anxiety and distress and therefore would be inappropriate for a reflective mental health app. Most agreed that the most viable funding option would be for the app to be funded by an institution such as schools, general practitioners (GPs) or the government, and then offered to students or patients. There was consensus that it would improve trust in the app if it was recommended by a ‘trusted’ institution:

*‘I don’t think I would pay for it because just because. Because there are like free services that you could use that I got by the equivalent to what you got on this app.’* UK

*‘I think if the university itself offered it, in the end you would find a lot of people interested.’* Spain

## Discussion

Using mixed methods, this research aimed to understand young people’s motivations for enrolling and participating in a mental health app trial, and the role of intrinsic and extrinsic drivers in motivating their engagement. Across all three trial arms, 21% of participants, after going through the consent process, never then set up an account to access the app and approximately 50% of participants who set up an account did not complete the 3-month follow-up assessment. Engagement was not significantly higher in the intervention arm compared to the control arms across primary adherence metrics (e.g. total number of intervention app tasks completed). Participants in the control group did use the app on significantly fewer number of days overall compared to the active intervention arms. Taken together with the qualitative interview findings, these results suggest that while extrinsic factors (e.g. financial incentive of up to £60 and undergraduate degree course credits) may prompt initial participation in research, intrinsic drivers (e.g. finding the app interesting and useful) may play a more critical role in fostering sustained engagement. However, this conclusion is based on a selective subsample of participants who completed the 3-month follow-up and engaged with the app to some degree. As such, these findings should be interpreted as preliminary and not necessarily representative of the broader participant population. Further research with more diverse and representative samples is needed to explore these dynamics in greater depth.

Consistent with findings from other app-based studies across health, sustained engagement has been problematic across app studies and in most populations.^10,15^ Research with adult populations suggests that financial incentives alone do not significantly increase app engagement and do not significantly affect users’ anxiety symptoms, depressive symptoms, well-being or emotion regulation difficulties,^33^ although financial pressures are likely to be different for student populations. Furthermore, some studies have had recruitment success into mental health app research without extrinsic incentives being offered. For example, the MindKind study was able to recruit over 1600 16- to 24-year-olds in the UK to participate in a mental health app-based study without any financial incentive offered.^34^ An additional intrinsic incentive might have been their contribution to knowledge and scientific enquiry as they were aware their data would be used to further our understanding and improve outcomes for others, as well as, potentially, for themselves. In the MindKind study, those with a history of mental illness were more likely to participate and median number of days participants were engaged in the app was 11 days, with 14% of recruits still engaged in the study at the 3-month follow-up.^34^

These findings are particularly important when considering the findings from any research that uses extrinsic incentives and how this then might inform future implementation and sustainability of mental health apps, particularly when they might then even apply a charge for the user to download and use (either one-off or subscription-base). Financially incentivising engagement in app-based mental health intervention research is therefore potentially problematic if young people participate for the money rather than because they are intrinsically motivated to do so, hence compromising our understanding of some key implementation drivers. Conducting research without financial reward may provide a better sense of young people’s engagement in ‘real-world’ circumstances to facilitate better dissemination and implementation.

### Limitations

In terms of the quantitative app engagement data, we did not control for baseline scores on the mental health measures in our analyses of engagement. For further details on the adherence criteria, levels of app engagement in both trials and trial outcomes please see eAppendix 4 in Watkins et al.^29^

Despite its strengths, the qualitative study was subject to several methodological limitations. Foremost, we were only able to gather data from participants who were randomised to the emotional competence app condition, who had downloaded the app, who had used the app at least once and who had completed the 3-month follow-up. This selective inclusion criteria ensured participants had sufficient exposure to the intervention to provide meaningful feedback; however, it also limits the generalisability of the findings to other subgroups, such as those who disengaged early or did not complete the trial. Despite our best efforts, we were unable to recruit trial participants who had never downloaded or used the app during the trial. We were therefore unable to explore barriers to engagement for the least engaged young people or understand why the app was not appealing to those who chose not to take part, despite having sufficient interest so as to click on the initial links. The views expressed by the participants in this study are therefore only representative of those who engaged with the app.

In addition, we did not interview any young people who had accessed the CBT version of the app as our primary aim was to understand the implementation drivers of the emotional competence condition. While some findings may be generalisable to other digital interventions, the exclusion of participants from other intervention arms further limits the scope of the qualitative findings. Finally, participants who agreed to participate in the qualitative study were financially incentivised to do so and often highlighted the importance of this incentive in keeping them engaged. Therefore, conclusions about naturalistic engagement, feasibility and acceptability of the app and prospect of free availability in schools, universities and health services or commercial availability are more difficult to make.

### Implications

Given the current limitations in our understanding of real-world app engagement, the way in which we conduct mental health app research may need to be re-examined. Early formative user testing on intervention design before randomised trials may help achieve improved intervention acceptability and feasibility, which can help support intrinsically incentivised engagement without the need for financial incentives. Similarly, pragmatic trials can help one to draw conclusions about the naturalistic engagement, feasibility and acceptability of apps. In addition, emerging evidence suggests that incorporating human support into digital mental health interventions can improve adherence and retention by addressing the diverse needs and preferences of users. For instance, periodic check-ins with clinicians, coaches or peers have been shown to enhance user motivation and engagement in digital interventions.^8,35^ Combining such human support with scalable app-based solutions could balance the benefits of personalisation with the scalability of digital tools, making interventions more effective and appealing to young people. Given that research findings consistently highlight how engagement patterns with apps are primarily short term, sustained engagement should be carefully considered before being a key outcome measure; it may be that a shift in focus is needed to more rapid single-session interventions, which are designed for brief engagement.^36–38^

In a market increasingly saturated with free, unregulated mental health apps, user-paid monetisation strategies are not likely to be components of a dissemination strategy as they are difficult to resource. An alternative solution is that the app is sponsored by health and educational institutions, as well as workplaces, who then can disseminate or prescribe access to patients, students and employees. The National Health Service (NHS) purchases subscriptions to a range of health apps, which are then prescribed to patients without charge.^39^ As well as reducing the financial burden on the user, being prescribed or recommended by a practitioner increases the likelihood of an individual using a mental health app.^40^

It is noted that commercial companies are currently collecting substantial data on multiple levels of engagement on mental health apps and our ability to better understand implementation drivers will be enhanced if these data are made available. Engagement data are currently difficult to access as they are rarely held on open access databases, preventing broader conclusions to be drawn for the field. Moving to a model similar to that for pharmacological study data, where all data are captured and appropriately shared, will potentially dramatically enhance our opportunities to learn about best ways to proceed.

Offering extrinsic incentives for participation in clinical trials might be thwarting progress, as data are potentially gathered on a population from which essential implementation information cannot then be gleaned. Moving to collecting data from as many sources as possible, alongside pragmatic trials addressing early implementation drivers, will ensure any opportunities mental health apps might provide are identified and disseminated.

## Data Availability

De-identified individual participant data and a data dictionary defining each field used for analysis will be made available with publication after approval of a proposal by the ECoWeB steering committee. Contact should be made with Edward Watkins (e.r.watkins@exeter.ac.uk).
